# A Deep Look into the Microbiology and Chemistry of Froth Treatment Tailings: A Review

**DOI:** 10.3390/microorganisms9051091

**Published:** 2021-05-19

**Authors:** Angeline Van Dongen, Abdul Samad, Nicole E. Heshka, Kara Rathie, Christine Martineau, Guillaume Bruant, Dani Degenhardt

**Affiliations:** 1 Northern Forestry Centre, Natural Resources Canada, Edmonton, AB T6H 3S5, Canada; angeline.vandongen@canada.ca; 2 Laurentian Forestry Centre, Natural Resources Canada, Québec City, QC G1V 4C7, Canada; abdul.samad@canada.ca (A.S.); christine.martineau@canada.ca (C.M.); 3 CanmetENERGY Devon, Natural Resources Canada, Devon, AB T9G 1A8, Canada; nicole.heshka@canada.ca (N.E.H.); kara.rathie@canada.ca (K.R.); 4 Energy, Mining and Environment Research Centre, National Research Council Canada, Montreal, QC H4P 2R2, Canada; guillaume.bruant@cnrc-nrc.gc.ca

**Keywords:** froth treatment tailings, diluent, microbial communities, GHGs, characterization, biodegradation

## Abstract

In Alberta’s Athabasca oil sands region (AOSR), over 1.25 billion m^3^ of tailings waste from the bitumen extraction process are stored in tailings ponds. Fugitive emissions associated with residual hydrocarbons in tailings ponds pose an environmental concern and include greenhouse gases (GHGs), reduced sulphur compounds (RSCs), and volatile organic compounds (VOCs). Froth treatment tailings (FTT) are a specific type of tailings waste stream from the bitumen froth treatment process that contains bioavailable diluent: either naphtha or paraffins. Tailings ponds that receive FTT are associated with the highest levels of biogenic gas production, as diverse microbial communities biodegrade the residual diluent. In this review, current literature regarding the composition, chemical analysis, and microbial degradation of FTT and its constituents is presented in order to provide a more complete understanding of the complex chemistry and biological processes related to fugitive emissions from tailings ponds receiving FTT. Characterizing the composition and biodegradation of FTT is important from an environmental perspective to better predict emissions from tailings ponds and guide tailings pond management decisions.

## 1. Introduction

Bitumen extraction from the Athabasca oil sands region (AOSR) plays an important role in meeting global and North American energy demands, however, the tailings produced during the extraction and treatment processes pose a variety of environmental concerns. For every 1 m^3^ of oil extracted from the oil sands, about 4 m^3^ of tailings waste are produced and stored in tailings ponds on site [1]. The total volume of fluid tailings in the AOSR is reported at over 1.25 billion m^3^ [2]. When residual hydrocarbons within the tailings biodegrade or volatilize, greenhouse gases (GHGs), reduced sulphur compounds (RSCs), and volatile organic compounds (VOCs) may be released [3]. Froth treatment tailings (FTT) are a specific type of tailings that contain bioavailable diluent, which is the primary source of GHG emissions from FTT tailings ponds and has been associated with localized bubbling within some tailings ponds [4,5,6]. Characterizing the composition and biodegradation of FTT is important from an environmental perspective to better understand how to reduce GHG emissions and manage the risks associated with tailings [7,8].

The purpose of this study is to present a review of current literature pertaining to FTT beginning with an overview of FTT generation and composition followed by an in-depth look at current methods used for determining the chemical composition of diluent, current research relating to the microbial biodegradation of FTT in tailings ponds, and recent findings regarding emissions from tailings ponds associated with diluent in FTT. This review presents current understandings pertaining to the complex chemistry and microbiology of FTT and highlights areas where further research is needed in order to address the complex challenge of quantifying and managing tailings pond emissions.

## 2. Background

**Figure 1:**
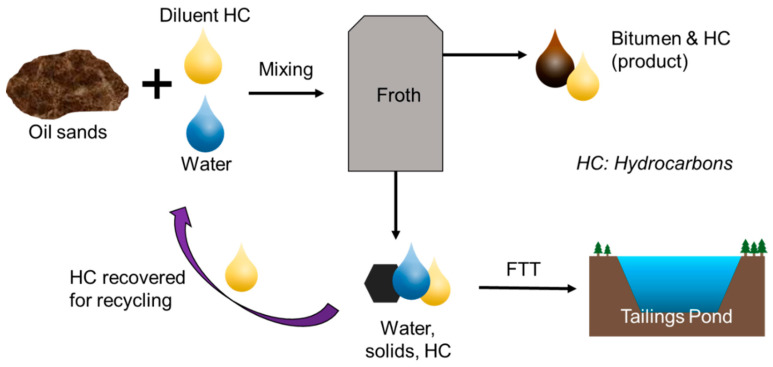
Primary separation process of bitumen from oil sands.

FTT are a waste stream of the oil sands extraction process. In order to extract useable hydrocarbons from oil sands deposits, the oil sands ore must first undergo treatment to separate the bitumen in oil sands from the water and sand. During primary extraction, oil sands are mixed with water, hydrocarbon diluent, and air to form a slurry [9]. Bitumen is then separated from the slurry by flotation, resulting in a bitumen-rich froth that also contains fine solids and emulsified water droplets [9,10]. During the subsequent froth treatment process, diluent is added to reduce the density and viscosity of the bitumen, thereby enhancing separation of the hydrocarbons from the solids and water [10]. These waste products become FTT. This extraction process is conceptually illustrated in Figure 1.

Two types of organic diluents are mainly used for froth treatment: naphtha and paraffinic solvents [11]. Naphtha refers to a mixture of C_5_–C_12_ aliphatic hydrocarbons and benzene, toluene, ethylbenzene and xylene (BTEX) compounds [12,13]. Paraffinic diluent is composed mainly of C_5_ and C_6_ alkanes [14]. The type of diluent chosen in the froth treatment process is based on the desired bitumen product quality [15]. Naphtha-based froth treatment has been predominantly used in the oil sands for over 30 years and typically requires a naphtha/bitumen ratio of 0.6–0.75 *w*/*w* [10,16]. The relatively newer paraffinic-based froth treatment process produces a cleaner bitumen product; however, this is achieved at a lower bitumen recovery rate and requires a higher diluent/bitumen ratio above 1.5 *w*/*w* [10]. After froth treatment, the diluent is recovered in solvent recovery units for reuse, but a fraction ends up in the FTT waste stream [6,11]. The exact chemical composition of FTT depends on the ore, extraction process, refining process, and additives used [3]. FTT are typically composed of 76.5 wt% water, 17 wt% mineral solid particles, 4.5 wt% bitumen, and up to 2 wt% diluent [10]. The mineral solid particles in FTT are primarily silicates with varying amounts of oxides, carbonates, sulphides, and sulphates [17]. FTT can also contain BTEX, polycyclic aromatic hydrocarbons (PAHs), hazardous metals, and naturally occurring radioactive minerals [4,17,18].

FTT are deposited in tailings ponds in the same ways as other types of extraction tailings: either subaqueously or subaerially onto the pond surface [14,16,17]. Solids and larger asphaltene components of the tailings settle quickly, forming a layer of sand and sediment on the beaches and bottom of tailings ponds, surrounding the fine clay particles, which are extremely slow to settle and are referred to as fluid fine tailings (FFT) [16,19]. Tailings are typically stored in ponds for years to allow for further dewatering and consolidation [20]. Once the fine tailings solid content reaches 30–40% *w*/*w*, these tailings are referred to as mature fine tailings (MFT) [19].

## 3. Types of Froth Treatment Tailings

### 3.1. Naphtha FTT

Naphtha is a complex mixture of low molecular weight *n*-, *iso*-, and cycloalkanes and monoaromatics (BTEX) [21,22]. Naphtha is produced during the bitumen upgrading process and is recovered for use as diluent [23]. Naphtha composition varies between operators; some use heavy naphtha containing mostly C_9_–C_16_ aliphatics, while others use light sour naphtha with mainly C_5_–C_8_, or naphtha containing primarily C_6_–C_10_ hydrocarbons [4,24]. An analysis of heavy naphtha found it contained 18 wt% *n*-alkanes, 31 wt% *iso*-alkanes, 27 wt% cycloalkanes and 15 wt% BTEX [6]. The residual naphtha concentration in tailings ponds from different operators ranges between 0.2 and 0.5 wt% [1,6,24,25]. After the bitumen extraction and naphtha recovery processes, the composition of residual naphtha released in FTT generally contains higher amounts of less volatile, heavier hydrocarbons [6].

Naphtha FTT may also contain small amounts of chemicals such as sodium hydroxide (NaOH), which is added during the extraction process to disperse clays, as well as demulsifier chemicals (surfactants), which are often added during froth treatment to help remove water droplets [14,16]

### 3.2. Paraffinic FTT

Compared to naphtha, paraffinic diluent is relatively simple and is composed primarily of C_5_ and C_6_ *n*- and *iso*-alkanes [8,22]. Analysis of paraffinic diluent found that it contained 24 wt% *n*-pentane, 11 wt% *n*-hexane, and 49 wt% *iso*-alkanes (2-methylbutane, 2-methylpentane, and 3-methylpentane) [26].

Paraffinic FTT composition differs from naphthenic FTT in a few other ways. Paraffinic solvents precipitate the asphaltene component of bitumen, which act as flocculants, further removing suspended solids and water droplets to achieve a cleaner bitumen product containing less moisture and solids [10,11]. The precipitated asphaltene aggregates are collected in the paraffinic FTT [10]. Additionally, paraffinic FTT contain trisodium citrate, which is added during the extraction process instead of NaOH, and polyacrylamide, which is added to thicken tailings before deposition in tailings ponds [14]. Trisodium citrate is an easily fermentable methanogenic substrate that may also contribute to tailings pond emissions [14,27].

## 4. Chemical Analysis of FTT Diluent

### 4.1. Diluent Extraction Techniques

As the biodegradation of residual hydrocarbons in tailings produces GHG emissions, the analysis and quantification of residual diluent in FTT is a critical step in understanding and predicting GHG emissions from tailings ponds. In order to directly examine the diluent in tailings, the sample is subjected to a solvent extraction to isolate the hydrocarbons in a nonaqueous matrix that is amenable to gas chromatography (GC) analysis. The extraction of diluent from tailings is complex due to the nonhomogenous nature of tailings, which contain water, solids, viscous hydrocarbons, and dissolved salts [28,29,30].

The efficiency of the solvent extraction relies on several important parameters, including subsampling technique, solvent selection, solvent ratio, agitation rate, and contact time [31,32]. The best practice for subsampling includes reducing particle size, homogenization, and random subsampling, in order to achieve results that accurately reflect the bulk sample [30]. Due to the volatile nature of diluents, it is also important to store and work with samples at 4 °C to prevent evaporative losses [29,33]. Solvent selection is an important consideration for diluent extraction. Solvents with similar polarity to that of the desired diluent components generally have good dissolving efficiencies [34]. Solvent ratio is considered the driving force for mass transfer [31]. Increasing the solvent ratio promotes interaction between the solvent and solute, and increases the concentration gradient between the liquid–solid phases [34]. Finally, agitation rate and contact time are important parameters for solvent extraction [31,32]. The contact time required to achieve full extraction depends on the level of turbulence, with lower turbulence levels requiring longer contact times [31].

It has been found that up to 30% of naphtha in tailings ponds can bind with bitumen [4]. Hence, to achieve accurate quantification of naphtha, bitumen would likely need to be extracted from the tailings sample as well. For bitumen extraction, solvents should have a combination of paraffinic and aromatic components to extract all components of bitumen, including the asphaltenes which are generally insoluble in paraffins, but partially dissolve in aromatic solvents [32,35]. Grimaldos et al. [36] reported higher diffusivity of bitumen into *n*-pentane compared to *n*-heptane or toluene due to its lower viscosity. For the extraction of bitumen from tailings containing large quantities of water, 1,1,1-trichloroethane has been used as the solvent to solubilize only the bitumen [30].

Hydrocarbons in mature fine tailings samples such as *n*-alkanes, naphtha, and BTEX have been extracted in several laboratory studies using organic solvents such as methanol and *n*-pentane to avoid dissolving the asphaltenes [6,25]. In Siddique et al.’s 2006 microcosm study, C_6_–C_10_ alkanes were extracted using a 10:1 solvent (methanol) to tailings ratio [1]. Siddique et al.’s 2007 study extracted naphtha from tailings using only 4 mL of solvent (*n*-pentane) for a 20 mL tailings sample [6]. For the extraction of analytes from tailings, mechanical/reciprocating shakers, sonification, or Soxhlet apparatuses have been used; however, shaking and sonication are much faster procedures than Soxhlet extraction [6,30]. The Syncrude naphtha extraction method requires five minutes of shaking, while Siddique et al.’s naphtha and alkane extraction procedures involved 10 and 30 min of shaking, respectively. Paraffinic diluent has been extracted from tailings samples using methanol as the solvent with a 10:1 solvent to tailings ratio, where samples underwent 30 min of shaking prior to settling and subsequent purge-and-trap GC analysis [22,26]. The use of different diluent types (paraffinic versus naphtha) should not have a significant impact on the extraction procedure, as both fall within a low to moderate carbon number range. The diluents will have similar considerations in terms of solubility and potential losses during handling.

### 4.2. Diluent Analysis Techniques

The measurement and characterization of the naphtha or hydrocarbon content remaining in tailings samples has been largely dominated by GC techniques. There is not a rich body of published literature to draw upon when trying to find an analysis method, particularly for FTT samples. Due to the heterogeneity of tailings samples, it can be difficult to use methods validated for one type of tailings on a second type. The works discussed in this section are methods applied to tailings matrices other than FTT but are the best place to approach an assessment of analysis techniques in the absence of other information. There are a number of publications regarding the measurement of methane in tailings samples. These methane methodologies can be used as a starting point for the quantification of higher hydrocarbons. Methane is commonly measured using headspace GC, where the sample is placed in a vial or other closed container and the methane emitted from the tailings is captured and measured [22,37,38,39]. Ongoing methane generation from incubation experiments has also been monitored over time using GC with thermal conductivity detection (TCD), and a packed stainless-steel column [40]. Tan et al. measured methane in cultures enriched from tailings via headspace GC with flame ionization detection (FID), with residual volatile hydrocarbons measured using GC-MS [41]. In some cases, a limited number of alkanes are measured by this headspace method, with GC-MS detection [21], or GC-FID detection [22].

A few specific methods exist in the literature for the analysis of diluent. Siddique and coworkers extracted *n*-alkanes, naphtha and BTEX from mature fine tailings (MFT) and analyzed the extracts using GC-MS and a DB5-MS column [25]. External standards and a quantitation program were used to measure specific compounds. A common approach to quantifying hydrocarbons or diluent in tailings samples is to use a method developed by the Canadian Council of Ministers of the Environment (CCME), which is an extraction and GC analysis protocol for petroleum hydrocarbons in soil [33]. This method produces data on hydrocarbons by four groups, F1–F4 (where F is an abbreviation for fraction), with each group containing hydrocarbons of increasing carbon number. Shahimin and Siddique demonstrate how these data can be used when analyzing paraffinic solvent hydrocarbons in MFT [24,26]. The F1 fraction from the CCME method was also used to approximate the naphtha remaining in MFT tailings samples, as the major components of naphtha fall within this fraction (C_6_–C_10_) [6]. To analyze individual components that may be present as a result of naphtha, tailings samples can be extracted, and subjected to PONAU analysis [6,24]. PONAU is an acronym that stands for paraffins, olefins, naphthenes, aromatics, and unknown components, and the analysis is known by a number of different similar acronyms, including PONA, PIONA and PIANO, with the additional “I” designating *iso*-paraffins [42]. PONAU uses a GC-FID system and retention time matching and Kovats indices to identify paraffins, olefins, naphthenes, aromatics and unknowns up to carbon number 15. Components are then binned into groups to give a mass percent value of each group. Given that the CCME fractions are a bulk grouping by carbon number, this breakdown of naphthenic components can provide valuable detail on extracted hydrocarbons.

## 5. FTT Microbial Diluent Degradation

### 5.1. Microbial Community Composition and Abundance in FTT

Tailings ponds harbor diverse indigenous microbial communities, which are actively involved in the biodegradation of residual hydrocarbons (naphtha, paraffins and bitumen) to release GHGs [14]. Tailings-associated microbes also play an important role in element cycling (sulphur, nitrogen and iron) and accelerating the consolidation of tailings [7]. The microbial populations in tailings originate primarily from the oil sands ore and process water, while airborne microbes may contribute to their assembly over time [14]. Each tailings pond harbors distinct microbial communities due to the unique tailings management practices of each operator, primarily during bitumen extraction and tailings consolidation processes. The management and processing history of legacy tailings ponds are not very well documented, thus making it challenging to decipher the type and quantity of tailings held in each pond [14]. Furthermore, the microbial community structure is primarily shaped by gradients of physiochemical properties in the stratified waterbody such as availability of O_2_, temperature, pH, tailings density and type of hydrocarbons in stratified tailings ponds [7,43].

The gradients of physiochemical properties along depth provide different niches for distinct types of microbial communities associated with tailings pond ecosystems. The surface water layer of tailings ponds is mainly aerobic due to wind and wave action, creating an oxygen gradient in underlying tailings zones. The sufficient availability of O_2_ in the uppermost layer creates favorable conditions for the aerobic catabolism of hydrocarbons. For instance, several bacterial taxa potentially capable of aerobically biodegrading hydrocarbons were detected in the surface layer of oil sands process water (OSPW) including *Pseudomonas*, *Flavobacterium*, and *Xanthobacter* [44]. Bacterial species belonging to *Methylocaldum* and *Methylomonas*, which are capable of aerobically consuming methane, were also found in fresh tailings pond water. Shotgun metagenomic sequencing identified particulate methane monooxygenase as a key enzyme in these methanotrophic communities [44]. Stasik et al. investigated elemental cycling and microbial activity as a function of depth and found vertical shifts in microbial biomass and activity in tailings ponds [45]. They found the highest biomass of aerobic thiosulphate-oxidizing bacteria compared to sulphate-reducing and iron-reducing bacteria at shallower depths (1–14 m) in tailings ponds.

**Figure 2:**
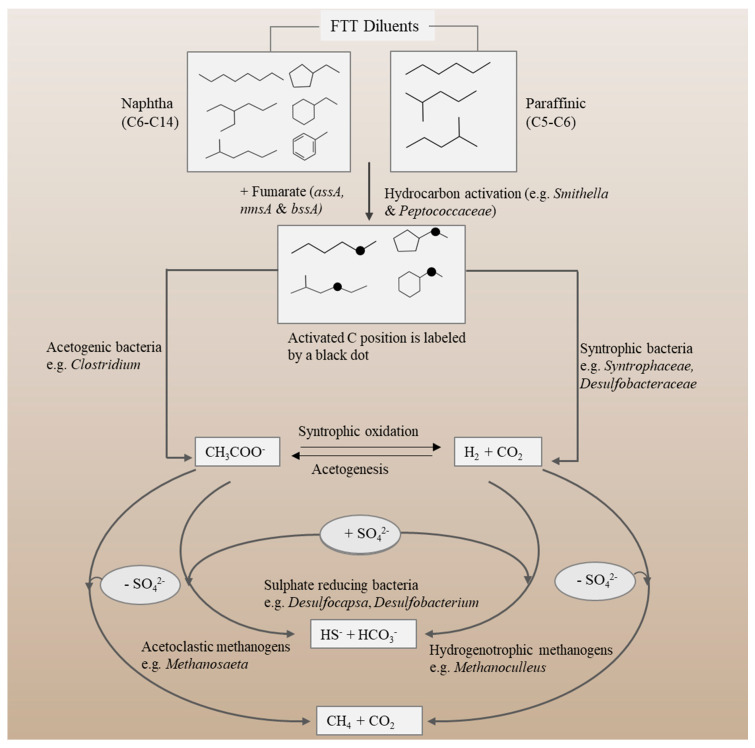
Principal microbial processes involved in the degradation of froth treatment tailings (FTT) diluents (naphtha and paraffinic). After substrate activation, syntrophic and/or acetogenic microorganisms degrade diluent hydrocarbon to simple compounds such as hydrogen (H_2_), carbon dioxide (CO_2_) and acetate (CH_3_COO^−^). Hydrogenotrophic and acetoclastic methanogens produce methane (CH_4_). The presence of sulphate (+SO_4_^2−^) or absence of sulphate (−SO_4_^2−^) can alter the competitive balance between sulphate reducers and methanogens. Diagram does not show some secondary products.

In contrast to surface layers, subsurface zones (FFT and MFT) in tailings ponds are mainly anoxic, containing mostly anaerobic microbial communities, which play key roles in methanogenesis, carbon turnover and elemental cycling. Methanogenic and sulphate-reducing microorganisms are dominant members of diverse anaerobic tailings communities, which mainly sustain on the diluent substrate arising from the FTT stream (Table 1 and Figure 2) [14,43]. Previous studies estimated the viable population (most probable number) of sulphate reducers (10^5^–10^9^ cells), nitrate reducers (10^9^ cells), iron reducers (10^3^ cells), and methanogens (10^2^–10^3^ cells) in one gram (dry weight) of mature fine tailings [14]. These subsurface anaerobic microbial communities utilize residual hydrocarbons from diluent and bitumen as carbon sources under redox conditions (carbon dioxide-reducing, sulphate-reducing and iron-reducing conditions) [7]. Core microbiome analyses of samples taken from six oil sands tailings ponds identified 2–5 operational taxonomic units (OTUs) that were shared by the majority of samples and that could be involved in hydrocarbon degradation. The core bacterial community comprised members of the *Betaproteobacteria* class (dominated by the *Comamonadaceae* family) involved in hydrocarbon degradation, denitrification, and iron reduction. An OTU belonging to the *Anaerolineaceae* family (phylum *Chloroflexi*) and involved in hydrocarbon degradation was also detected. Among the methanogenic community, *Methanosaeta* was found dominant and was shared by most samples. However, the majority of OTUs was identified as part of a diverse accessory microbiome potentially involved in nutrient cycling and the transformation of organic fractions of tailings [46]. The quantity and type of diluent is the main driver of anaerobic microbial activities in tailings ponds that receive FTT [4].

Using pyrosequencing of samples from a gypsum-treated tailings pond, Ramos-Padrón et al. [40] detected shifts in microbial communities as a function of depth. They identified sulphate-reducing bacteria (*Desulfurivibrio* and *Desulfocapsa*), syntrophs (*Smithella*, *Pelotomaculum* and *Syntrophus*) and both acetoclastic and hydrogenotrophic methanogens (*Methanosaeta*, *Methanolinea* and *Methanoregula*) in deeper zones, while specific taxa belonging to *Brachymonas*, *Cellulomonas* and *Thiobacillus* were only detected in the deepest layers of tailings ponds. Although anaerobic microbial communities are common in the subsurface zones of oil sands environments [14,50], one study surprisingly found a high abundance of aerobic bacterial taxa particularly belonging to *Burkholderiales* (e.g., *Cupriavidus*), *Rhizobium*, *Brevundimonas*, *Methylobacterium*, *Pseudomonas* and *Acinetobacter* in subsurface samples of oil sands, which may indicate the probability of oxygen ingress into the subsurface layers [51]. Additionally, it may show the capability of specific bacterial strains adapting to anoxic conditions (e.g., *Pseudomonas*, *Acinetobacter*) [52,53]. Indeed, some strains of the *Pseudomonas* and *Acinetobacter* genera have been shown to sustain anaerobic conditions [52,53].

### 5.2. Key Microbial Species Involved in Diluent Biodegradation

It has been widely accepted that diluent hydrocarbons (naphtha and paraffins) are the primary carbon sources for indigenous anaerobic microbial communities in tailings ponds that receive FTT streams [4,7,14]. Many studies (Table 1) used components of naphtha diluent to decipher structure and functions of tailings-residing microbial communities [25,43,49]. Recently, Siddique et al. assessed the microbial community structure in MFT amended with naphtha (*iso*- and cycloalkanes) and found significant shifts in the microbial community structure during diluent metabolism [43]. A dramatic increase of 45% in the bacterial family *Peptococcaceae* was observed in the amended culture after 815 days of incubation. At the genus level, *Desulfotomaculum* was dominant, representing more than 95% of the *Peptococcaceae*. The archaeal community structure was quite stable, with the exception of the *Methanomicrobiaceae* family, which increased after 815 days of incubation [40]. When MFT from Albian and CNRL ponds were spiked with paraffinic solvent to study microbial community composition, 16S rRNA gene pyrosequencing revealed dominance of *Peptococcaceae* and *Anaerolineaceae* in the bacterial community and *Methanosaetaceae* in the archaeal community [26]. Taken together, bacterial families *Peptococcaceae* (*Clostridia*), *Syntrophaceae* (*Deltaproteobacteria*), *Anaerolineaceae* (*Anaerolineae*) (Table 1) were found frequently enriched in diluent-spiked tailings under anoxic conditions [22,25,26,43,49]. However, enrichment of distinct microbial taxa during certain types of diluent biodegradation revealed that structure of hydrocarbons is an important driver of anaerobic microbial community structure in tailings ponds. For instance, *Peptococcaceae* showed a preference for shorter (C_5_–C_6_) alkane biodegradation and *Syntrophaceae* were more inclined towards longer chain alkanes (C_6_–C_18_) metabolism. However, MFT spiked with monoaromatics (BTEX) and *n*-alkanes (C_6_–C_10_) showed enrichment of taxa from the *Peptococcaceae*, *Syntrophaceae* and *Anaerolineaceae* families. Likewise, hydrocarbon structure-based preferences were observed in the methanogenic archaeal community. Acetoclastic methanogens (mostly *Methanosaetaceae*) were enriched ubiquitously in tailings containing short-chain *n*-alkanes diluents, while co-occurance of acetoclastic and hydrogenotrophic methanogens (*Methanosarcinales* and *Methanomicrobiales*) was observed in the tailings amended with relatively complex diluent hydrocarbons (*iso*-alkanes or longer-chain *n*-alkanes) [7,14,43].

### 5.3. Biodegradation Pathways

The biodegradability of different naphtha components, such as *n*-alkanes, *iso*-alkanes, and BTEX by endogenous methanogenic microbes in tailings ponds has been demonstrated by several laboratory studies using tailings pond samples spiked with individual hydrocarbons [1,21,22,25,41]. Mohamad Shahimin and Siddique spiked tailings pond samples with 0.2 wt% naphtha and reported up to 52% reduction in naphtha, including complete biodegradation of *n*-alkanes and the majority of *iso*-alkanes after a long incubation time (1600 days) [24]. A pattern of preferential biodegradation of *n*-alkanes followed by *iso-* and cycloalkanes has been observed [24,43]. Within the *n*-alkane fraction of naphtha, Mohamad Shahimin and Siddique [24] found nC_6_ and nC_7_ were the preferred metabolites; however, previous studies found a contradictory preference for larger carbons (C_10_ > C_9_ > C_8_ > C_7_), perhaps due to the octanol/water partitioning coefficient increasing with chain length [1,6]. The preferential sequence of BTEX degradation by methanogenic bacteria is toluene > *o*-, *m-* and *p*-xylene > ethylbenzene > benzene [6].

The methanogenic biodegradation of paraffin and its major constituents has also been studied in laboratories using spiked tailings samples from different operators [26,49]. Mohamad Shahimin and Siddique observed an 81% reduction in paraffinic solvent after a 1300-day incubation in Shell Albian tailings [26]. Complete biodegradation of *n*-alkanes and 2-methylpentane and partial degradation of 2-methylbutane and 3-methylpentane was reported, indicating a preference for *n*-alkanes over *iso*-alkanes, which was also observed in the biodegradation of naphtha [24,26].

Biodegradation of hydrocarbons under methanogenic environments takes place in a series of steps (Figure 2), which require an intimate syntrophic relationship between fermentative bacteria and methanogenic archaea [54]. Indigenous tailings microbial communities can readily degrade major fractions of diluents (e.g., *n*-alkanes and *iso*-alkanes), while certain complex hydrocarbons (cycloalkanes) may be recalcitrant to biodegradation [14,43]. Hydrocarbon-degrading bacteria utilize various alternative enzymatic reactions for activation of the substrate. Different oxygen-independent activation pathways of hydrocarbons have been proposed, including hydroxylation, carboxylation, fumarate addition, reverse methanogenesis, and water addition at multiple bonds [55]. However, enzymatic addition of hydrocarbons to fumarate has been a widely reported mechanism under various anoxic conditions [7,55,56]. The glycyl radical enzymes (GREs) catalyze the addition of hydrocarbons to fumarate to yield aromatic-substituted succinates. Among the GREs family, benzylsuccinate synthase (BSS) is an intensely studied enzyme which catalyzes the first step in anaerobic toluene degradation to produce benzylsuccinate. These activation reactions result in ring saturation, β-oxidation, and finally yielding benzoyl-coA [55]. The GREs can activate the C–H bond in a wide range of hydrocarbons (such as toluene, cresols, cyclohexane, ethylbenzene, xylenes, methylnaphthalene, and *n*-alkanes) through attachment to fumarate [54,55]. All GREs share the principal mechanism with BSS. The putative GRE alkylsuccinate synthase activates alkane by adding hydrocarbons across the double bond of fumarate to form alkyl-substituted succinates [54,55]. The enzyme alkylsuccinate synthase plays a key role in catalyzing fumarate addition reaction under sulphate- and nitrate-reducing conditions [57]. Tan et al. found homologues of putative succinate synthase genes (*assA*, *nmsA* and *bssA*) associated with the fumarate addition pathway of hydrocarbon activation under methanogenic environment [58]. Moreover, the fumarate addition pathway and genes encoding alkylsuccinate synthase (*assA*, *assB*, *assC*, and *masE*) were detected in versatile methanogenic alkane-degrading cultures [21,41], including members of *Smithella* and *Peptococcaceae* [59,60].

After activation of the substrate, diluent hydrocarbons are further degraded by hydrogenotrophic and/or acetoclastic methanogens (Figure 2). Hydrogenotrophic methanogens such as *Methanocella*, *Methanococcus*, *Methanobacterium* and *Methanothermobacter* utilize H_2_ as an electron donor during the process of CO_2_ reduction into methane. In the acetoclastic pathway, archaea such as *Methanosaeta* primarily use acetate as the terminal electron acceptor for methane production. Generally, both acetoclastic and hydrogenotrophic methanogenesis are an integral part of methane-producing pathway in oil sands tailings ponds. However, dominance of methanogenic populations of one or the other pathway may depend on several factors, including principal concentrations of metabolites (acetate, hydrogen, and formate), and also the availability of electron donors and acceptors, CO_2_ concentrations, temperature, pH, salinity, availability of nutrients, permeability and porosity [7,54,56].

Overall, diluent biodegradation under methanogenic conditions requires diverse microbial communities for initial anaerobic activation of substrate, followed by conversion of degradation products by syntrophic communities and final conversion by archaeal methanogens to produce CO_2_ and methane [54,56].

## 6. Fugitive Emissions from FTT Tailings Ponds

### 6.1. Greenhouse Gases (GHG) Generation Rates

Significant amounts of biogenic GHGs are emitted from tailings ponds as microbial communities biodegrade available hydrocarbons into CH_4_ and CO_2_ [8]. According to a compilation of emission data taken between 2010 and 2011 from major oil sands tailings ponds, Syncrude’s Aurora In-Pit is the largest emitter of CO_2_ at 498 t/ha/year and Syncrude’s Mildred Lake Settling Basin (MLSB) is the largest emitter of CH_4_ at 26 t/ha/year [3]. The global warming potential of CH_4_ is approximately 25 times that of CO_2_, making it a potent greenhouse gas and powerful contributor to global warming [61].

CH_4_ was first observed bubbling from Syncrude’s MLSB in the 1990s after 15 years of operation [5,62]. It was estimated that 2–5% of the fine tailings volume was CH_4_, a portion of which was escaping to the atmosphere at rates exceeding 10 g CH_4_/m^2^ in some areas [5]. Furthermore, the area of the pond that initially began bubbling was associated with receiving naphtha FTT [14]. Alternatively, Suncor’s Aurora Settling Basin, which does not receive FTT, reported no CH_4_ emissions when sampled in 2011–2012 [4]. It has since been demonstrated that tailings ponds that receive FTT are associated with higher levels of GHG emissions [3]. Residual diluent provides a better substrate than bitumen for stimulating methanogenesis, leading to the production of CH_4_ and CO_2_ [24,63].

#### 6.1.1. GHG Generation in Studies Using Spiked Samples

Several microcosm studies have been conducted involving tailings samples from various operators spiked with different hydrocarbon substrates and incubated under anaerobic conditions. In an early effort to determine the methanogenic substrate in tailings, tailings samples from Syncrude’s MLSB were spiked with increasing concentrations of bitumen, but increased CH_4_ production was not detected, suggesting that the residual bitumen may not be the source of methanogenesis in tailings [63]. In 2006, Siddique et al. spiked tailings samples from Syncrude’s MLSB with a mixture of short-chain *n*-alkanes (C_6_–C_10_) and recorded CH_4_ production within one week, indicating that the *n*-alkanes in diluent are capable of sustaining methanogenesis in tailings ponds [1]. Other diluent constituents, including *iso*-alkanes, longer chain *n*-alkanes (C_14_–C_18_), some cycloalkanes, and some BTEX components, were also shown to contribute to CH_4_ production in spiked microcosm studies [6,21,25,43]. Based on results from these studies, a first order kinetic model was developed and used to estimate a maximum potential yield of 280 m^3^ CH_4_/ton of pure naphtha [64].

In 2017, Gee et al. amended tailings samples with 0.2, 0.8, and 1.5% *w/v* naphtha diluent in anaerobic mesocosms and evaluated the emissions over an 11-week period. Production of CH_4_ and CO_2_ was observed mostly after five weeks (two weeks after sulphate depletion) and increased with increasing diluent concentrations, up to a maximum of 40.7 µmol CH_4_/mL tailings and 5.9 µmol CO_2_/mL measured at 1.5% *w/v* naphtha [65]. The higher amount of CH_4_ compared to CO_2_ was contradictory to most tailings pond emissions reports, but was attributed to a lack of methanotrophs in the anaerobic mesocosms, which are normally found consuming CH_4_ in upper layers of tailings ponds [3,65].

Other microcosm studies have compared the methanogenic potential of endogenous microbe communities between different tailings ponds, including Syncrude MLSB, CNRL Horizon (which both receive naphtha FTT), and Shell Albian (which receives paraffinic FTT) amended with either naphtha or paraffinic diluent (or their constituents) [22,24,26,49]. In all of these studies, similar masses of CH_4_ were recorded between microcosms, demonstrating the metabolic flexibility of tailings microbial communities in biodegrading both diluent types; however, there was some variation in the lag times preceding methanogenesis. Siddique et al. [49] found that the Shell Albian microcosms amended with paraffin components had a longer lag time than the Syncrude tailings, perhaps because the Albian pond has not been exposed to FTT for as long (~10 years) as Syncrude’s MLSB (~35 years). Comparisons between Shell Albian and CNRL Horizon tailings found that the CNRL Horizon tailings amended with paraffinic solvent had the longest lag time before methanogenesis [22,26].

These microcosm studies also illustrate the differences in CH_4_ emissions produced based on diluent type. Shell Albian and CNRL Horizon tailings spiked with paraffinic diluent biodegraded 81% and 59% of the diluent, respectively; however, in another study where the same tailings were spiked with naphtha diluent only 52% and 45% of the diluent was biodegraded over the same time period [24,26]. As naphtha diluent contains more complex and recalcitrant compounds, a smaller portion of the diluent is biodegraded, resulting in less CH_4_ produced compared to the same quantity of paraffinic diluent over a 1600 day experiment [26]. This difference was observed in Mohamad Shahimin and Siddique’s microcosm studies, where similar amounts of CH_4_ were produced from tailings spiked with 0.2 wt% naphtha and 0.1 wt% paraffinic diluent [24,26]. The difference in GHG emissions increases further considering that higher diluent/bitumen ratios are required for paraffinic froth treatment process [15].

#### 6.1.2. GHG Generation in Studies Using Field Samples

Although most studies have focused on methanogenesis in microcosms amended with hydrocarbons/diluent, unamended microcosms can provide information about the methanogenic potential of endogenous substrates within the tailings. Holowenko et al. evaluated CH_4_ released from unamended microcosms incubated for around 500 days and reported that most total yields were between 0.10 and 0.25 mL CH_4_/mL, regardless of incubation temperature, showing that methanogenesis is a finite process dependent on substrate availability [5]. Unamended Syncrude MLSB and Suncor Pond 1 tailings microcosms contained 2% *v/v* CH_4_ after 15 days and 1% *v/v* after 30 days, respectively [37]. In several similar microcosm studies, the total quantity of CH_4_ produced by the unamended control microcosm containing 50 mL of tailings was between 0.005 and 0.008 mmol/mL, representing CH_4_ from endogenous substrates in the tailings samples [1,6,49].

Siddique et al.’s 2020 microcosm study evaluated CH_4_ production at different tailings depths and reported higher CH_4_ yields (3.6 mmol over 630 days) in unamended tailings from a shallower depth of 6 m compared to deeper unamended tailings from 31 m (0.03 mmol over 1550 days) [43]. The difference was attributed to higher concentrations of labile hydrocarbons in the younger shallower tailings [43].

To compare the effects of residual naphtha in FTT with fresh naphtha on methanogenesis, Siddique et al. [6] amended tailings microcosms with 50% FTT from Syncrude’s Plant 6 (equivalent to ~0.25% residual naphtha), and compared these results with microcosms spiked with 0.2% pure naphtha. After 36 weeks, 2.8 mmol of CH_4_ was produced from the FTT amendment, compared to 4.4 mmol of CH_4_ from 0.2% pure naphtha [6]. These results support the idea that pure naphtha loses some of its lighter components through volatilization and dissolution into bitumen during processing, resulting in residual naphtha in FTT with less preferred substrates for methanogens [64]. Additionally, diluent in tailings can become incorporated into bitumen globules, making it less available for biological degradation [66].

#### 6.1.3. Factors Impacting GHG Generation Rates

The microbial community composition in tailings ponds will influence the type of methanogenesis (acetoclastic or hydrogenotrophic) that will occur, which in turn dictates production rates and proportions of CH_4_ and CO_2_ emitted [67]. As such, factors that impact microbial activity, such as type and availability of carbon sources, electron donors, temperature, and toxicity are important to consider in terms of GHG emissions [67]. Residual diluent from FTT provides a labile carbon source to anaerobic microbes, making diluent concentration the primary contributor to GHG emissions from tailings ponds [1,4,65,66]. Availability and type of electron acceptors such as sulphate and bicarbonate can also influence microbial communities [67]. Research has shown that SRB out-compete methanogens for the same substrates; therefore, sulphate levels need to be sufficiently depleted before methanogenesis can occur [1]. Tailings temperature will also influence emission rates as methanogens tend to have increased activity at higher temperatures [5]. A study found that the same quantity of CH_4_ was produced faster at 22 °C compared to the typical tailings temperature of 14 °C [5]. GHG emission rates are likely higher during the summer months, where temperatures over 20 °C have been recorded in the upper layers (0–5 m) of tailings ponds [68]. High concentrations of toxic compounds, such as BTEX or naphthenic acids also impact microbial activity and thereby may inhibit methanogenesis [6,67]. The presence of methanotrophs in upper layers of tailings ponds may also reduce CH_4_ emissions from ponds as aerobic methanotrophic bacteria oxidize CH_4_ into CO_2_ [44].

Another factor influencing tailings pond GHG emissions is age; older ponds emit higher CH_4_ concentrations, which is associated with lengthy lag periods prior to methanogenesis and older ponds having more established microbial communities [3,22]. However, it has also been noted that the settling of mature fine tailings in ponds over time could reduce GHG emissions as biogenic gases may become entrapped in the settling particles, or possibly retained under increased hydraulic pressure [64,66].

Finally, the movement and size of gas bubbles formed within the pond can affect GHG emission rates, as large CH_4_ bubbles are more likely to successfully travel to the pond surface where they are released to the atmosphere [27]. A column study using Shell Albian paraffinic tailings found that the largest bubbles were observed in columns treated with citrate, polyacrylamide, and diluent [27].

The emission of GHGs from tailings ponds is a complex issue, and although models have been developed to estimate emissions based on diluent concentrations, they are based mostly on laboratory studies, which do not capture the extent of heterogeneity and complexity found in tailings ponds [66].

### 6.2. Reduced Sulphur Compounds (RSCs)

Reduced sulphur compounds (RSCs) are a group of chemicals from natural and anthropogenic sources that contain sulphur atoms in their lowest oxidation state (S^−2^) and are generally characterized by strong odors [69]. The most abundant RSCs in the environment include H_2_S, carbonyl sulphide, methane thiol, dimethyl sulphide, carbon disulphide, and dimethyl disulphide [63]. In the atmosphere, the cycling of RSCs can lead to acidic precipitation and increased aerosol particles [70]. The effects of RSCs on human health and vegetation are diverse and depend on the specific compound and exposure level [69]. H_2_S is known to be highly toxic at low concentrations and can be fatal above 500 ppm [71].

Oil sands tailings contain SRB that use available organics, such as diluent, as electron donors to reduce sulphate into dissolved sulphides, which can escape as H_2_S [72]. Emissions from numerous oil sands tailings pond management areas were tested in 2015, and all reported to emit either less than detectable or very low (<0.0031 t/ha/y) levels of H_2_S [3]. An analysis of a sample from Syncrude’s West In-Pit tailings reported low H_2_S levels of 0.01 mM and acid volatile sulphur, chromium reducible sulphur, and dimethylformamide extractable sulphur concentrations of 0.38, 0.31, and 0.21 mg/g dry weight of tailings, respectively [73]. In Salloum et al.’s study, where tailings mesocosms were amended with sulphate, only 3% of the added sulphate was detected as dissolved sulphides [62]. The low levels of H_2_S found in these field and laboratory studies has been attributed to sulphur being precipitated into metal sulphides, sulphur being cycled within ponds, and dissolved sulphides being mostly in the form of HS^−^ at the slightly alkaline pH of tailings ponds [3,62,72,73]. As such, H_2_S emissions from tailings ponds may become a concern if the pH is low or if sulphide production exceeds the amount of available metals [62,65]. It has also been suggested that H_2_S produced at depth may partition into rising CH_4_ bubbles, carrying it to surface where it is chemically and biologically oxidized to sulphate [40,72]. Furthermore, the oxidation of sulphide to sulphate is likely the cause of higher levels of sulphate reported in the surface waters of tailings ponds [5,40,72]. In a study where sulphide was incubated with tailings and 20% *v/v* oxygen, complete sulphide oxidation was observed after 100 min and was only slightly slower in autoclaved tailings, indicating that sulphide oxidation is a primarily chemical process [40].

In Gee et al.’s 2017 mesocosm study, where tailings samples were amended with naphtha diluent, it was reported that sulphur reduction rates and H_2_S production increased with diluent concentration, demonstrating that diluent stimulates SRB in the tailings [65]. Interestingly, other RSCs, including 2-methylthiophene, 3-methylthiophene, 2,5-dimehtylthiophene, thiofuran, and butyl mercaptan, were detected in the controls that did not contain tailings and increased with increasing diluent, indicating that these RSCs likely originated from the naphtha diluent itself [65]. Overall, the total RSC production rates after 6 weeks were between 0.01 and 0.02 µmol RSC/mL tailings with H_2_S and 2-methylthiophene making up 81% of the RSCs produced [65].

To accelerate the densification of tailings, some operators, such as Suncor and Syncrude, add gypsum (CaSO_4_·2H_2_O) to their tailings at around 1 kg/m^3^ as part of the tailings treatment process to form composite tailings (CT) or centrifuge tailings (CF) [14,37,40]. Gypsum aids the release of water from the tailings slurry, resulting in high concentrations of sulphate (>1000 mg/L) in pore water and released water [37]. Increased sulphate concentrations from gypsum may also play a role in reducing GHG emissions. When sulphate is abundant, the reduction of sulphate into sulphide inhibits the reduction of CO_2_ into CH_4_, as SRB out-compete methanogenic archaea for the same substrates, principally H_2_ [5,37,40,74]. Fedorak et al. [37] observed that CH_4_ was not detected in tailings samples until sulphate concentrations dropped to around 20 mg/L.

### 6.3. Volatile Organic Compounds (VOCs)

VOCs are compounds of carbon (excluding CO and CO_2_) which evaporate under normal atmospheric conditions and participate in atmospheric photochemical reactions [75]. In Canada, VOCs are classified as a toxic substance in the Environmental Protection Act, and numerous studies have confirmed their toxicity, mutagenicity, and carcinogenicity [76,77]. VOCs enter the lower atmosphere where they can harm human health, reduce air quality, and pollute the surrounding environment [18,78]. Through a series of atmospheric chemical reactions, VOCs contribute to the formation of tropospheric ozone and secondary organic aerosols, which account for a large portion of particulate matter <2.5 µm in the atmosphere [79,80].

Air samples collected during a flight over the AOSR found that 70% of C_2_–C_10_ VOC compounds analyzed were significantly enhanced over the oil sands region compared to the local background, with *n*-heptane and *n*-octane (components of naphtha diluent) reaching 397× and 242× the background levels, respectively [80]. It was deduced that a portion of the VOCs (C_4_–C_9_ alkanes, C_5_–C_6_ cycloalkanes, C_6_–C_8_ aromatics) was related to evaporation from oil sands products and/or diluent [80]. In a report of VOC emission fluxes based on in-situ measurements from 19 different tailings management areas, VOC emission rates ranged from 0.01 to 14.60 t/ha/y, averaging around 1.75 t/ha/y [3].

Although VOCs are not produced by microorganisms, they are another important source of emissions from tailings ponds receiving FTT. Tailings ponds that receive FTT generally have higher reported VOC emissions [3]. When FTT are discharged into tailings ponds, some of the volatile hydrocarbons in the diluent are released into the air [78]. It has been found that 40% of naphtha diluent in tailings contributes to VOCs [29]. BTEX, a component of naphtha diluent, is one of the most commonly emitted VOCs from most tailings ponds [3]. A preliminary study using tailings samples spiked with naphtha found that between 20% and 60% of the naphtha evaporated, depending on the clay and bitumen content [81]. A strong interaction between naphtha and bitumen was indicated by decreased naphtha volatilization with increasing bitumen content [81]. In Burkus et al.’s 2014 GHG model, it was assumed that 30–35% of naphtha is volatilized and 40% of lighter paraffinic diluent is volatilized.

## 7. Final Remarks

In summary, FTT are a type of tailings generated by the bitumen froth treatment process that contain either naphthenic or paraffinic diluent. The biodegradation of diluent in FTT is the primary contributor to biogenic emissions from tailings ponds. Tailings ponds that receive FTT streams generally have higher GHG emissions, VOC emissions, and higher potential to generate RSCs depending on microbial community, tailings composition, and pond physical properties. Although tailings ponds harbor dynamic microbial populations that are highly diverse due to tailings characteristics, some bacterial and archaeal taxa, notably *Peptococcaceae* and *Methanosaetaceae*, have been found consistently in various studies regardless of diluent type and incubation time. Microbial inhabitants of tailings can degrade large fractions of residual diluent. Simple hydrocarbons are preferably metabolized first, followed by relatively complex hydrocarbons and finally more complex and recalcitrant hydrocarbons. Diluent degradation takes place in a series of steps involving a syntrophic partnership between fermentative bacteria and methanogenic archaea through hydrogenotrophic and/or acetoclastic pathways. The biodegradation of diluent and its constituents by microbial communities in tailings has been well studied in laboratory experiments, however, several areas require further research in order to better understand the complex chemical and microbial process contributing to emissions from tailings ponds receiving FTT.

In most of the laboratory studies, tailings samples were spiked with pure diluent, whereas the actual residual diluent concentration and composition in FTT is variable and less well studied. The analysis of diluent remaining in tailings samples, particularly FTT, is a critical step in determining the effect of hydrocarbons on the microbial activity in tailings ponds and associated GHG emissions. While a somewhat patchy body of literature exists regarding the extraction and analysis of hydrocarbons, a comprehensive study of solvents, extraction conditions, and GC parameters is missing. When approaching analysis of FTT, we must rely on techniques that were developed for different tailings types. A systematic study of extraction parameters for FTT would yield the most accurate data and ensure that data is not being biased by using ill-suited conditions.

Predicting GHG, RSC, and VOC emissions from tailings ponds is difficult due to the heterogeneity and complexity within tailings ponds. Further research is needed to confirm predicted VOC losses associated with different diluent types. For assessing GHG emissions from distinct diluent hydrocarbons, stoichiometric models have been developed, but they are not well supported by field data. Until now, most studies have relied on the pyrosequencing method of DNA sequencing, however, advanced sequencing techniques with more in-depth coverage is vital to understand the vast complexity of tailings microbes. Additional research is required to capture the spatial and temporal patterns of FTT-associated microbial activity to better predict GHGs and guide tailings pond management decisions.

## Figures and Tables

**Table 1:** Key microbial taxa enriched during diluent degradation.

Diluent Type	Diluent Hydrocarbon	Tailings Source	Archaea	Bacteria	Sequencing Method	* References
Naphtha	*n*-alkanes (C_6_–C_10_), BTEX, whole naphtha (Syncrude)	MLSB (Syncrude)	*Methanosaetaceae* and *Methanomicrobiales*	*Clostridiales* and *Syntrophobacterales*	Clone library	[47] ^w^
	*iso*- and cycloalkanes (C_6_–C_10_)	MLSB (Syncrude)	*Methanosaeta*, *Methanoregula* and *Methanoculleus*	*Peptococcaceae* and *Smithella*	Pyrosequencing	[43]
	*iso*-alkanes (C_7_–C_8_)	MLSB (Syncrude)	*Methanoregula* and *Methanosaeta*	*Peptococcaceae*	Pyrosequencing, cloning and T-RFLP	[21]
	*n*-, *iso*- and cycloalkanes (C_6_–C_10_)	MLSB (Syncrude)	*Methanosaetaceae* and *Methanomicrobiaceae*	*Peptococcaceae*	Pyrosequencing	[41]
	toluene (^13^C_6_–^12^C_7_)	MLSB (Syncrude)	*Methanosaeta*	*Clostridiales* (*Desulfosporosinus*), *Desulfobulbaceae*	T-RFLP	[48]
	*n*-alkanes (C_14_–C_18_)	MLSB (Syncrude)	*Methanomicrobiales* (*Methanoculleus*) and *Methanosarcinales* (*Methanosaeta*)	*Syntrophaceae* (*Syntrophus*)	Clone libraries	[25]
	*n*- *iso*- and cycloalkanes (C_6_–C_10_), whole naphtha (CNRL)	MRM (Albian) and Horizon (CNRL)	*Methanosaetaceae* and *Candidatus Methanoregula*	*Anaerolineaceae*, *Syntrophaceae* and *Peptococcaceae*	Pyrosequencing	[24] ^w^
Paraffinic	*n*- and *iso*-alkanes (C_5_–C_6_)	Syncrude and Albian	*Methanosaeta*, *Methanoregula* and *Methanolinea*	*Peptococcaceae*	Pyrosequencing	[49]
	*n*-, *iso*- and cycloalkanes (C_5_–C_6_)	MRM (Albian) and Horizon (CNRL)	*Methanosaetaceae* and *Candidatus Methanoregula*	*Anaerolineaceae* and *Peptococcaceae*	Pyrosequencing	[26]

MLSB = Mildred Lake Settling Basin, MRM = Muskeg River Mine, CNRL = Canadian Natural Resources Ltd., T-RFLP = terminal restriction fragment length polymorphism. *All studies used an artificial mixture of commercially available hydrocarbons (diluent components) as diluent except the studies labeled “^w^” which used whole naphtha received from mining operators.

## Data Availability

Not applicable.
